# Practical Surgery

**Published:** 1839-01

**Authors:** 


Art. XI.—*Practical Surgery: with one hundred and thirty Engravings on Wood.* By Robert Liston. *Second Edition.—London,* 1838. 8vo. pp. 529.

So short a period has elapsed since we noticed Mr. Liston's work on its first appearance, (*Brit. and For. Med. Review,* No. X. p. 457,) and the opinion of the profession, evinced by an almost immediate call for a new edition, has so unequivocally confirmed the favorable judgment we then pronounced on it, that it is unnecessary to dwell on the character or contents of the volume before us. We may, however, state that several additions and alterations have been made, and a few new woodcuts added in the present edition; so that our present report of the work, if at all different from that formerly made, must be only more favorable. It is a volume which no young surgeon should be without.

